# Behavioral effects of targeted drug delivery via non-invasive microbubble enhanced focused ultrasound blood brain barrier opening in non-human primates

**DOI:** 10.1186/2050-5736-3-S1-P16

**Published:** 2015-06-30

**Authors:** Matthew Downs, Amanda Marie Buch, Maria Eleni (Marilena) Karakatsani, Carlos Sierra Sanchez, Shangshang Chen, Vincent Ferrera, Elisa Konofagou

**Affiliations:** 1 Columbia University, New York, New York, United States

## Background/introduction

The Blood Brain Barrier (BBB) in Non-Human Primates (NHP) can be non-invasively opened through the use of Focused Ultrasound (FUS) in conjunction with microbubbles. This procedure allows for a targeted, transient opening in the BBB of the NHP which can be utilized to facilitate drug delivery. While FUS has been used to deliver various pharmacological compounds to promote neurogenesis or treat cancer, no group has investigated if drug delivery can affect behavioral responses. In this study, we show the effects of targeted ME-FUS drug delivery on the responses of NHP to a decision making task.

## Methods

The ME-FUS procedure was conducted on three (n = 3) NHP under general anesthesia (isoflurane) and in stereotactical positioning to ensure accurate targeting.

Microbubbles (4-5 um, in-house prepared) were administered IV at the onset of applying the FUS (Single element transducer, 500 kHz, 0.3-0.4 Mpa, 10 ms pulse length, 120 second duration). The putamen region of the basal ganglia was targeted for all behavioral experiments. After the ME-FUS procedure there was a three hour recovery period allowing the NHP to fully recover normal motor functions impaired from the general anesthesia.

IM injections of haloperidol (D2 antagonist, 0.01mg/kg) in the awake monkey were administered five minutes prior to the start of behavioral testing. The NHPs were trained to complete a reward magnitude bias and dot direction coherence task for water reward. One day after the ME-FUS procedure BBB opening and safety of the procedure was verified with contrast enhanced T1-weighted and T2-weighted MRI scans respectively.

## Results and conclusions

The BBB was successfully opened in the putamen region of each NHP for all ME-FUS procedures. No damage from the ME-FUS procedure was detected with the T2-weighted MRI scans. Delivery of haloperidol after BBB opening was shown to nullify a significant difference of reaction time between the contralateral and ipsilateral hands to the ME-FUS procedure observed with the saline controls. This significant difference in reaction time between hands was observed when haloperidol was administered without BBB opening. Delivery of haloperidol after BBB opening also negated a significant difference in reaction time between the high and low reward cues observed with the saline controls. Overall preliminary results show that FUS-induced BBB opening in NHP can be used to facilitate delivery of D2 antagonists to the putamen region causing significant effects on motivation and reaction time.

